# Root morphology and anatomy respond similarly to drought and flooding in two wheat cultivars

**DOI:** 10.1093/aob/mcaf152

**Published:** 2025-07-22

**Authors:** Tobias Guhr, Zhiwei Song, Albert G Andersen, Juan de la Cruz Jiménez, Ole Pedersen

**Affiliations:** Department of Biology, University of Copenhagen, Universitetsparken 4, 3rd floor, Copenhagen 2100, Denmark; Institute for Cellular and Molecular Botany, University of Bonn, Regina-Pacis-Weg 3, Bonn 53113, Germany; Department of Biology, University of Copenhagen, Universitetsparken 4, 3rd floor, Copenhagen 2100, Denmark; Department of Biology, University of Copenhagen, Universitetsparken 4, 3rd floor, Copenhagen 2100, Denmark; Department of Biology, University of Copenhagen, Universitetsparken 4, 3rd floor, Copenhagen 2100, Denmark; Department of Biology, University of Copenhagen, Universitetsparken 4, 3rd floor, Copenhagen 2100, Denmark; School of Biological Sciences, The University of Western Australia, 35 Stirling Highway, Crawley, WA 6009, Australia

**Keywords:** Aerenchyma, drought, flooding, porosity, radial water loss, root morphology, *Triticum aestivum* L

## Abstract

**Background and Aims:**

Wheat (*Triticum aestivum* L.) is widely grown in regions prone to both drought and flooding conditions. Although root responses to drought and flooding have been extensively studied separately, studies comparing key anatomical root traits in wheat in both conditions side-by-side are rare. We tested the hypothesis that wheat roots respond in a similar manner to both drought and flooding, despite these being contrasting water regimes.

**Methods:**

Two wheat cultivars (‘Jackson’ and ‘Frument’) were grown hydroponically in control conditions, drought and flooding, and the responses in plant growth, root morphology, root anatomy, development of apoplastic barriers and their capacity to reduce radial water loss were measured.

**Key Results:**

Xylem-to-stele ratio decreased by 33% under water stress compared with control conditions, whereas aerenchyma-to-cortex ratio increased 2.1–fold during both drought and flooding compared with control conditions. Compared with control conditions, lateral root growth was more reduced than adventitious root growth, 86% and 67%, respectively, under both types of water stress. There was comparably stunted root and shoot growth under water stress, and adventitious roots grew slower likewise and to one-third of length compared with control conditions. Our findings did not indicate differences in soil flooding tolerances between the two cultivars.

**Conclusions:**

We conclude that different underlying physical processes during contrasting water regimes, e.g., water limitation during drought and oxygen deficiency during flooding, result in similar root responses, e.g., increased relative aerenchyma area, lignin and suberin deposition in the endodermis, and decreased lateral-to-adventitious root length. Future research should provide a more comprehensive understanding of cross-stress effects on root morphology, anatomy and physiology.

## INTRODUCTION

In the 2024–25 growing season, 793.8 Mt of wheat (USDA, 2025) are projected to be harvested globally; however, water stress owing to water deficiency (i.e., drought) or excess (i.e., flooding) will limit the growth and productivity (Zampieri *et al.*, 2017; Mbow *et al.*, 2022). Advantageous root acclimatizations to cope with these contrasting soil environments include maximizing water uptake or conserving limited water in drought conditions (Aroca *et al.*, 2012), whereas root traits that facilitate internal aeration and prevent toxic compounds from entering the root from the flooded soil appear more crucial in soil flooding conditions (Pedersen *et al.*, 2021; Peralta Ogorek *et al.*, 2024). Understanding the functional benefits of key root traits that develop in drought and provide advantages in flooding conditions, or vice versa, could inform breeding programmes aimed at developing wheat cultivars with outstanding performance in both drought and flooding scenarios.

In terms of root morphology, roots grow steeply into the soil to access moist soil layers during drought, but total root length still declines in comparison to well-watered conditions (Liu *et al.*, 2004; Anjum *et al.*, 2017; Zhou *et al.*, 2018). The allocation of resources to below-ground organs, which fosters steep root growth, results in an increase in the root–to–shoot ratio during drought. In contrast, the growth of wheat roots and shoots slows down during flooding, partly owing to root tip death during anoxia, and this leads to a strong reduction in the root–to–shoot ratio (Herzog *et al.*, 2016). Wheat roots grown in soil under drought reduce the resource cost of elongation by reducing root diameter and thus increasing surface area relative to volume, whereas thicker roots under flooding improve tolerance to anoxic conditions, possibly owing to more space for aerenchyma formation in combination with a lower surface areas relative to volume (Yamauchi *et al.*, 2014; Steinemann *et al.*, 2015).

Inducible aerenchyma refers to large gas–filled spaces in the cortex originating from ethylene–induced programmed cell death and is a common response of plants to both drought and flooding conditions (Lynch, 2018; Yamauchi *et al.*, 2018; Yamauchi and Nakazono, 2022). During drought, aerenchyma formation reduces root metabolic costs for root growth (Lynch, 2018), whereas under flooding, aerenchyma increases longitudinal oxygen diffusion from the shoot to the root tip (Xu *et al.*, 2013; Herzog *et al.*, 2016). Tissue porosity, which includes both aerenchyma and intercellular air spaces, was reported to increase from 5.2% to 14.8% under flooding in adventitious roots in 17 genotypes of wheat (Herzog *et al.*, 2016).

On an anatomical scale, tissue ratios are key traits that reflect adaptation or acclimatization to contrasting soil water regimes. A recent field study of 18 Poaceae species that are adapted to a range of soil water contents found that common features of wetland Poaceae species in their natural habitat include higher aerenchyma–to–cortex ratio (ACR), cortex–to–stele ratio (CSR) and xylem–to–stele ratio (XSR) (Yamauchi *et al.*, 2021). The CSR increases during flooding, and it is hypothesized that increased space for aerenchyma and oxygen diffusion is beneficial (Peralta Ogorek *et al.*, 2024). In contrast, there was no response in CSR when well–watered rice (*Oryza sativa* L.) was subjected to drought instead of flooding. In the same study, XSR decreased under both water stresses, probably reducing the risk of cavitation during drought (Peralta Ogorek *et al.*, 2024 ).

Suberization and lignification of cell walls of the endodermis is another key example of similar responses to both drought and flooding in wheat. During water stress, there is not only more intense deposition of suberin and lignin, but also an earlier onset of biopolymer deposition (Suresh *et al.*, 2024). A lignified hypodermis (referred to as exodermis in the study by Xu *et al.*, 2013) can occur in wheat adventitious roots during flooding. A later study could not confirm lignification in the hypodermis anywhere except directly below the root–shoot junction, which is also the case in other Poaceae species, and wheat is not known to form an exodermis, unlike rice (Ouyang *et al.*, 2020). Apoplastic barriers of suberin and lignin formed during water stress, in addition to anatomical acclimatizations, such as increased root diameter, can decrease radial water loss from the root to the surrounding media, as demonstrated in rice (Song *et al.*, 2023, 2024).

The objective of this study was to characterize wheat root responses to both drought and flooding side by side in hydroponic culture, thus further elaborating on previous submergence studies on these genotypes. We hypothesized that wheat roots respond in a similar manner to both drought and flooding, despite these being contrasting water regimes, because many of the root traits that confer tolerance to soil flooding are also relevant during drought, as suggested by Song *et al.* (2023) and Peralta Ogorek *et al.* (2024). We imposed drought and flooding by altering the composition of the nutrient solution on the two wheat cultivars, ‘Jackson’ and ‘Frument’, and measured key morphological, anatomical and physiological root traits after 3 weeks of treatment. We found that drought mirrored the effects of flooding on root length and root tissue composition. We conclude that different underlying physical processes during contrasting water regimes, e.g., water limitation during drought and oxygen deficiency during flooding, result in similar root responses, e.g., increased relative aerenchyma area, lignin and suberin deposition in the endodermis, and decreased lateral-to-adventitious root length.

## MATERIALS AND METHODS

### Plant growth and treatment

Seeds of *Triticum aestivum* L. ‘Jackson’ and ‘Frument’ were used in a previous study in combination, where ‘Frument’ emerged more sensitive than ‘Jackson’ to submergence, hence we could build on existing knowledge (Herzog *et al.*, 2018). The seeds were imbibed for 3 h in aerated 0.5 mm CaSO_4_ solution and pre–germinated in Petri dishes on a wet paper towel that was soaked in 0.5 mm CaSO_4_ solution for 2 days at room temperature in the dark. Seedlings were then grown on floating mesh in 3 L pots in 25% aerated nutrient solution for 9 days (see below for composition of full-strength solution). The plants were transferred to 5 L pots and fixed with foam in holes in the lids. Both the pots and lids were covered on the outside with aluminium foil to prevent algal growth via light from reaching the nutrient solution. The plants in the 5 L pots were grown for 3 weeks in full-strength nutrient solution containing 1.5 mm CaSO_4_, 7.5 mm MES, 0.4 mm MgSO_4_, 3.75 mm KNO_3_, 0.625 mm NH_4_NO_3_, 0.2 mm KH_2_PO_4_, 0.1 mm Na_2_O_3_Si, 0.05 mm Fe–EDTA, 50 µm KCl, 25 µm H_3_BO_3_, 2 µm MnSO_4_, 2 µm ZnSO_4_, 0.5 µm CuSO_4_, 0.5 µm Na_2_MoO_4_ and 1 µm NiSO_4_, with additives depending on the three treatments. The pH was adjusted to 6.5 with 2 m potassium hydroxide. The nutrient solution was continuously aerated to simulate well–watered control conditions. We used aerated nutrient solution with 10% (w/v) PEG–6000 for a high osmotic nutrient solution to mimic drought (−0.14 MPa) (Song *et al.*, 2023) and deoxygenated, stagnant nutrient solution with 0.1% (w/v) agar to imitate soil flooding (Wiengweera *et al.*, 1997), with the nutrient solutions being replaced weekly. The experiment was conducted in a completely randomized design with three treatments, two genotypes and five pots per treatment. Pots were treated as replicates, and unless otherwise stated, a replicate is defined as a randomly selected plant of a certain cultivar from a pot. Plants were grown in a greenhouse at 28 °C and long–day conditions (16 h light–8 h dark) with artificial lighting (500 µmol m^−2^ s^−1^) in Denmark (55°42′N, 12°33′E) between November and December 2024.

### Root extension

The length of a randomly selected emerging adventitious root (initial length ∼12 mm) was measured by marking it with a fine string near the root base and taking photographs of the root every second day between 10 and 18 days of treatment (DOT). The photographs were analysed using ImageJ software (National Institutes of Health, Bethesda, MD, USA).

### Root morphology

Five plants per cultivar were harvested at 0 DOT to provide initial values, and five plants per cultivar and treatment were harvested after 21 DOT. Root systems were spread and scanned at 600 DPI using an Epson Expression 13000XL flatbed scanner with a transparency unit (Epson, Suwa, Japan). Images were analysed using RhizoVision Explorer v.2.0.3 software (Seethepalli *et al.*, 2021). Lateral roots were defined as having a diameter range of ≤0.5 mm, and adventitious roots were defined as having a diameter range of >0.5 mm. The values at 0 DOT were averaged and subtracted from the values at 21 DOT to exclude root growth prior to stress commencement. The shoot and root systems were then dried in an oven at 60 °C to constant weight. The dry mass of the shoots and root systems was measured with a four–digit balance, the mean dry mass of the initials was subtracted, and the root–to–shoot ratio was calculated.

### Root anatomy

Roots 10–12 cm long from control plants and 8–9 cm long from stressed plants were sampled after 20 DOT and stored in 1.6% (v/v) paraformaldehyde phosphate–buffered saline solution. Root cross–sections were prepared at the base (0–10 mm from the root–shoot junction) and the tip (30–40 mm behind the root tip) using a vibrating microtome (Leica VT1200S, Leica Biosystems). Cross-sections of ∼100 µm thickness were cut at a speed of 1 mm s^−1^ and an amplitude of 1 mm and stored in deionized water. For analysis of relative tissue areas and areal ratios using ImageJ software, images of unstained cross–sections were taken in brightfield using an epifluorescence microscope (Nikon ECLIPSE Ci) with an attached camera (Nikon DS–Fi3, auto–exposure and white balance). Lignin in cross–sections was stained brown–red by incubating the cross-sections in 5% (w/v) phloroglucinol solution and 20% (w/v) HCl for ≥3 min each prior to brightfield microscopy as described above. Suberin in endodermal cell walls of cross–sections was stained by incubating the sections in 0.01% (w/v) Fluorol Yellow 088 solution for ≥1 h. Suberization was evident as a yellow–green fluorescence observed under ultraviolet light (excitation filter Ex 365/10, dichroic mirror DM–400 and barrier filter BA–400). To prevent differences in bleaching, cross–sections were exposed to ultraviolet radiation for 3 s before imaging with 10 ms exposure time.

### Radial water loss

Radial water loss (RWL) from roots to a dry atmosphere has recently been identified as a key indicator of internal tissue barriers to radial water movement (Song *et al.*, 2023). The RWL was measured following a method used by Peralta Ogorek *et al.* (2021) with some modifications. Adventitious roots were cut 6–7 cm behind the tip and lateral roots removed. The weight of 150–250 mg adventitious root tissue was monitored in a closed five–digit balance chamber in a <30% relative humidity atmosphere for 1 h by using silica gel grains in the weighing chamber. The length and diameter of root segments were measured using ImageJ software. Tissue was dried at 50 °C to constant weight and weighed. Root segment length, diameter and dry mass were included in the calculations to determine cumulative water loss. The RWL was calculated from these parameters, with values standardized to a root diameter of 1 mm to account for the non-linear relationship between root diameter and surface area-to-volume ratio, which gives rise to contrasting RWL because thin roots will desiccate faster than thick roots (Song *et al.*, 2023).

### Root respiration

Root tissue respiration was measured by quantifying oxygen consumption with an oxygen optode (Opto–MR, Unisense A/S), according to Winkel *et al.* (2013). Root segments were cut 25 mm behind the tip and placed in a 4 mL glass vial filled with deionized water at air equilibrium. The root segments rested for 15 min to allow for wound healing. The optode was calibrated with an oxygen-free solution (0.5 m NaOH and 0.5 M l–ascorbate) and air-equilibrated deionized water prior to measurements. The temperature was maintained at 25 °C. During the measurements, the optode was moved between the vials in a fixed order for four rounds. Each round took 10 min. The oxygen consumption rates (in nanomoles of O_2_ per second) of in–between rates from Unisense software (SensorTrace Suite v.3.2) were multiplied by the vial volume and divided by the fresh mass of root tissue to obtain the final respiration rates (in nanomoles of O_2_ per second per gram fresh mass). Unlike rice, wheat does not form an outer apoplastic barrier that limits radial oxygen loss (and therefore also uptake) (Perumalla *et al.*, 1990; Liu and Kreszies, 2023), hence we assumed free oxygen diffusion between the medium and root tissue.

### Tissue porosity

Root tissue porosity was assessed using the buoyancy method as described by Pedersen *et al.* (2009). Adventitious roots were cut 10 cm from the base and lateral roots removed. The porosity of fresh adventitious root tissue (300–700 mg) was determined by weighing the fresh weight and weight of tissue when it was buoyant in deionized water with a four-digit balance. The buoyant measurement was repeated after 15 min of vacuum infiltration with deionized water such that all gas-filled spaces were filled with water. Porosity was calculated as the percentage gas volume per unit tissue volume (Pedersen *et al.*, 2009), as established by Thomson *et al.* (1990).

### Photosynthetic parameters

Net photosynthesis was measured on the second youngest fully expanded leaf of the main tiller with the LI–COR 6800 portable gas exchange system (LI–COR Biosciences Inc., Lincoln, NE, USA). Environmental parameters were controlled during measurements to match the greenhouse conditions: 500 µmol s^−1^ air flow rate; leaf vapour pressure deficit of 1; 850 µmol mol^−1^ CO_2_ and 600 µmol m^−2^ s^−1^ light (90/10 red/blue). Measurements were conducted with 3 min of acclimation in the period between 09:00 and 13:00 h from 0 to 18 DOT.

### Statistical analysis and visualization

The datasets were assessed for normality using the Shapiro–Wilk test (α = 0.05) and, if necessary, transformed to meet the assumptions of parametric tests. Two–way ANOVA (α = 0.05) and Tukey’s HSD test for normally distributed data or the Kruskal–Wallis and Dunn’s test for skewed data that could not be transformed successfully were conducted in RStudio v.2022.12.0. The figure legends and Table 1 provide additional information on the details of the data transformation and statistical tests. Data were visualized in Graphpad Prism v.8.3.0 software and graphs and photographs arranged in Affinity Publisher 2 v.2.5.7 software (PANTONE LLC, 2023).

**Table 1.** Key root trait versus source of variation, percentage of total variation explained by each variable, and the *P*-value for two-way ANOVA.

Variable	Source of variation	Percentage of total variation	*P*-value
Root length ratio Figure 1	Cultivar	1.9	n.s.
	Treatment	62.2	<0.0001
	Cultivar × treatment	0.3	n.s.
	*Residual*	35.6	
Adventitious root length Figure 1	Cultivar	0.1	n.s.
	Treatment	77.2	<0.0001
	Cultivar × treatment	3.2	n.s.
	*Residual*	19.5	
Root dry mass Figure 1	Cultivar	0.2	n.s.
	Treatment	80.9	<0.0001
	Cultivar × treatment	2.0	n.s.
	*Residual*	17.0	
Radial water loss (RWL_50_) Figure 4	Cultivar	37.4	0.0001
	Treatment	11.1	n.s.
	Cultivar × treatment	9.8	n.s.
	*Residual*	41.7	
Respiration Figure 4	Cultivar	2.4	n.s.
	Treatment	19.8	n.s.
	Cultivar × treatment	6.0	n.s.
	*Residual*	71.8	
Cortex-to-stele ratio cv. ‘Jackson’ Figure 2	Position	33.2	0.0002
	Treatment	14.3	0.0287
	Position × treatment	10.8	n.s.
	*Residual*	41.6	
Cortex-to-stele ratio cv. ‘Frument’ Figure 2	Position	58.8	<0.0001
	Treatment	16.5	<0.0001
	Position × treatment	7.6	0.0122
	*Residual*	17.1	
Xylem-to-stele ratio cv. ‘Jackson’ Figure 2	Position	1.9	n.s.
	Treatment	69.7	<0.0001
	Position × treatment	5.7	n.s.
	*Residual*	22.7	
Xylem-to-stele ratio cv. ‘Frument’ Figure 2	Position	34.3	<0.0001
	Treatment	37.5	<0.0001
	Position × treatment	9.1	0.0093
	*Residual*	19.1	
Aerenchyma-to-stele ratio cv. ‘Jackson’ Figure 2	Position	13.1	0.0052
	Treatment	49.6	<0.0001
	Position × treatment	4.1	n.s.
	*Residual*	33.2	
Aerenchyma-to-stele ratio cv. ‘Frument’^1^ Figure 2	Position	n.a.	n.s.
	Treatment	n.a.	0.0016
	Position × treatment	n.a.	0.0004

Non-significant *P*-values > 0.05 are not shown (n.s.).

^1^Percentage of total variance is not available (n.a.) because the Kruskal–Wallis test was used.

## RESULTS

### Diminished growth in response to water stress

**Fig. 1.**
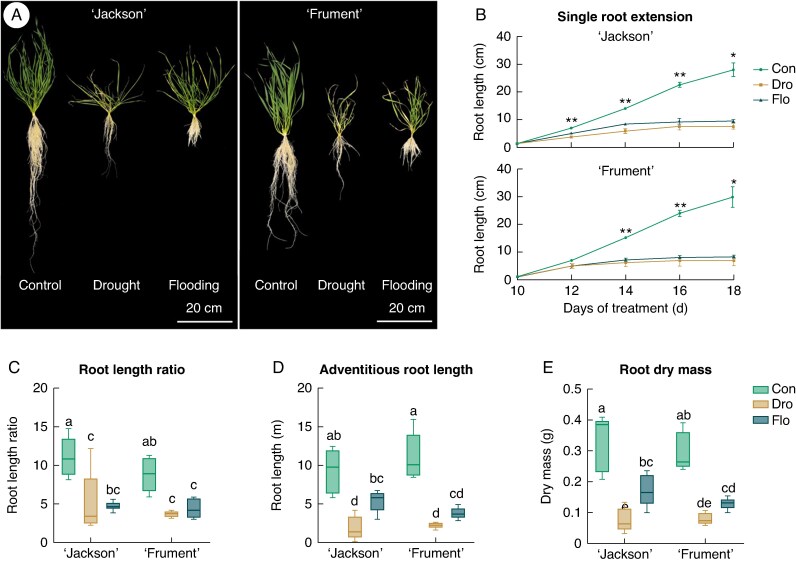
Habitus photographs (A), root extension (B), ratio of lateral to adventitious root length (C), adventitious root length (D) and root dry mass (E) of two wheat cultivars (‘Jackson’ and ‘Frument’) grown in control, drought or flooding conditions. (A) Photographs were taken after 23 days of treatment and the background removed using Affinity Photo 2 v.2.5.7. (B) The root extension of single emerging adventitious roots was measured between 10 and 18 days of treatment. Values are means and SE (*n*_‘Jackson’_ = 4, *n*_‘Frument’_ = 3). **Significant difference between controls and both drought and flooding; *significant difference between controls and drought only (Tukey’s *post hoc* test). (C, D) Roots with a root diameter of <0.5 mm were classified as laterals and >0.5 mm as adventitious roots (*n* = 5 root systems). In C, lateral root length (Supplementary Data Fig. S1B) was divided by adventitious (D) root length. In E, the root dry mass formed during the treatments is shown. Treatments were imposed on 9-day-old seedlings, and to account for the root growth prior to commencing the treatments, samples of each cultivar (*n* = 5) were harvested before treatments and the mean values of root dry mass were subtracted from values of samples from final harvest. (C–E) Tukey’s *post hoc* tests (α = 0.05) were performed, with different letters showing significant differences. See Table 1 for details on two-way ANOVA results. The box-and-whisker plots display the median (horizontal line), the first and third quartiles (box), and the minimum and maximum values (whiskers). Abbreviations: Con, control; Dro, drought; Flo, flooding.

We found that growth impairment of the root system and green tissue during water stress was evident visually (Fig. 1A). When monitoring individual adventitious root extension, we found similarly strong growth impairment in both drought and flooding starting 2 days after the first measurement, but with no differences between cultivars (Fig. 1B). Under water stress, adventitious roots reached their full length within 4–6 days of growth, whereas control roots continued to grow throughout the 8 days of measurement. Freshly emerged adventitious roots from controls grew to a length of 28.9 cm within 8 days, whereas in flooding and drought conditions they reached an average maximum length of only 9.1 and 7.3 cm, respectively. The initial growth rate under water stress was lower compared with controls and, assuming a linear growth rate during the first 2 days, the controls grew 2.9 cm day^−1^, compared with 1.7 cm day^−1^ under drought and 1.9 cm day^−1^ under flooding.

Using hydroponics, we recovered the whole root system, scanned it and calculated the length and surface area using RhizoVision Explorer. Total adventitious and lateral root lengths were highly correlated (*r* = 0.96; Pearson correlation analysis; Supplementary Data Fig. S1) and both declined significantly under water stress; however, drought had a slightly stronger effect than flooding (significant only for ‘Jackson’) (Fig. 1D; Supplementary Data Fig. S2A). There was no significant effect of cultivar on adventitious or lateral root length (Table 1). We found a significant effect of treatment, but no effect of cultivar, on the ratio of lateral to adventitious root length, which decreased strongly under water stress compared with controls (Fig. 1C). The lateral root length was 10–fold greater than adventitious root length in control conditions, whereas this ratio was only 4.3–fold under drought and 4.5–fold under flooding. This response was also reflected in the surface area of lateral versus adventitious roots (Supplementary Data Fig. S2B).

Root dry mass (DM), shoot DM and root–to–shoot ratio were negatively affected by water stress, and the response was more pronounced under drought than flooding (Fig. 1E; Supplementary Data Fig. S2D, E). Two–way cultivar × treatment ANOVA showed a significant effect only of treatment on root DM and shoot DM (Table 1); the two cultivars responded similarly to water stress. Root DM was reduced by 77% under drought and by 52% under flooding. The decline in shoot DM was also stronger in drought conditions, with the root-to-shoot ratio being significantly different only in ‘Jackson’ in drought conditions.

### Water stress–induced aerenchyma formation, suberization and lignification of the endodermis

**Fig. 2.**
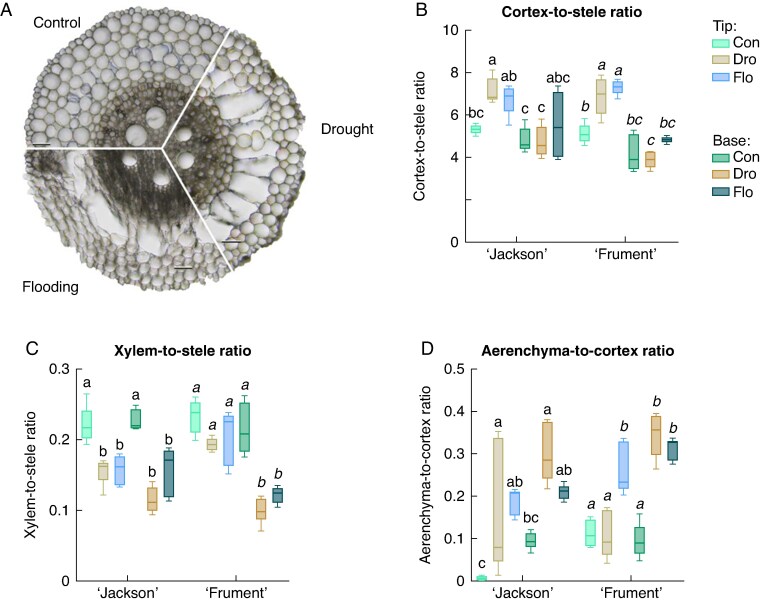
Typical adventitious root cross-sections (A), cortex-to-stele ratio (B), xylem-to-stele ratio (C) and aerenchyma-to-cortex ratio (D) of adventitious root cross-sections from the base and the tip. Two wheat cultivars (‘Jackson’ and ‘Frument’) were grown in control, drought or flooding conditions, and adventitious roots were sampled for analysis after 20 days of treatment. The two positions represent younger (30–40 mm the root tip) and older (base region, 0–10 mm behind the root––shoot junction) tissue. Tissue ratios were calculated from the same cross-sections (*n* = 5) using ImageJ software. Tukey’s *post hoc* tests were performed for each cultivar separately, with different letters representing significant differences. See Table 1 for details on two-way ANOVA results. To evaluate the effects of position and treatment on the aerenchyma-to-cortex ratio of ‘Frument’, we used a two-step non-parametric approach (Kruskal–Wallis test, followed by Dunn’s *post hoc* test). The box-and-whisker plots display the median (horizontal line), the first and third quartiles (box), and the minimum and maximum values (whiskers). Abbreviations: Con, control; Dro, drought; Flo, flooding.

We examined the response of cultivars on root tissue ratios and relative tissue area to water stress within tissues of the same age, because these traits are known to respond to soil flooding and drought conditions (Fig. 2A). We also compared these traits between younger tissue at the root tip and older tissue at the root base, assuming that tissue at each position was of the same age across treatments. A two–way cultivar × treatment ANOVA revealed no significant effect of cultivar on cortex-to-stele ratio (CSR), xylem-to-stele ratio (XSR) or aerenchyma-to-cortex ratio (ACR) in either younger or older tissues. We then tested interactions between tissue age and treatment to detect stress–induced changes in longitudinal gradients (Table 1). There was a significant effect of tissue age on CSR, relative cortex area and relative stele area in both cultivars, and of treatment on CSR, according to a two-way position × treatment ANOVA (Fig. 2B). There were no significant differences in older root tissues between controls and water stress. The CSR in younger tissues increased by 25% under drought and 26% under flooding compared with controls, which was attributable to increased relative cortex area (‘Jackson’, 4% and ‘Frument’, 5% compared with controls) to the detriment of relative stele area (Fig. 2B; Supplementary Data Fig. S3C, D). Root cross–sectional area was similar across all treatments in younger and older tissues, indicating a trade–off between relative cortex and stele area (Supplementary Data Fig. S3A).

Two-way position × treatment ANOVA showed a significant effect of treatment on XSR and relative xylem area in both cultivars. Relative xylem area was significantly lowered under water stress compared with controls, regardless of tissue age or cultivar (Supplementary Data Fig. S3D). Therefore, XSR declined by 51% under drought and 47% under flooding compared with controls in older tissue (Fig. 2C). In younger tissues, XSR decreased in ‘Jackson’ by 29% under drought and flooding, but was similar to controls in ‘Frument’. This was attributable to a significantly decreased relative stele area under water stress in younger tissues in ‘Frument’, which elevated XSR.

Two–way cultivar × treatment ANOVA and Tukey’s *post hoc* test revealed that there was a significant effect of cultivar on ACR in older tissue and that ACR was significantly higher in ‘Frument’ compared with ‘Jackson’ in older tissues under flooding. We performed a two-way position × treatment ANOVA and found a significant effect of treatment in both cultivars and a significant interaction effect in ‘Frument’. There was a significant increase (4–fold) in ACR between younger and older tissue under drought in ‘Frument’. In general, ACR increased notably under water stress compared with controls regardless of tissue age and cultivar (Fig. 2D). The ACR was mainly influenced by aerenchyma formation and not by cortex area (Supplementary Data Fig. S3B, E). In younger tissue of ‘Jackson’, there was a large variation in ACR under drought. These findings on root anatomy were supported by measurements of tissue porosity of root segments, which include both aerenchyma and intercellular air spaces (Supplementary Data Fig. S4). Under water stress, when aerenchyma formed, tissue porosity increased from an average of 8% in controls to 10% under drought and 14% under flooding. In summary, our results demonstrate that water stress induced consistent anatomical shifts within root tissues, notably increasing the CSR and ACR, particularly in younger tissues. Although cultivar differences were limited, stress-induced reductions in XSR and enhanced tissue porosity were observed across treatments, highlighting common strategies of root anatomical adjustment to both drought and flooding.

**Fig. 3.**
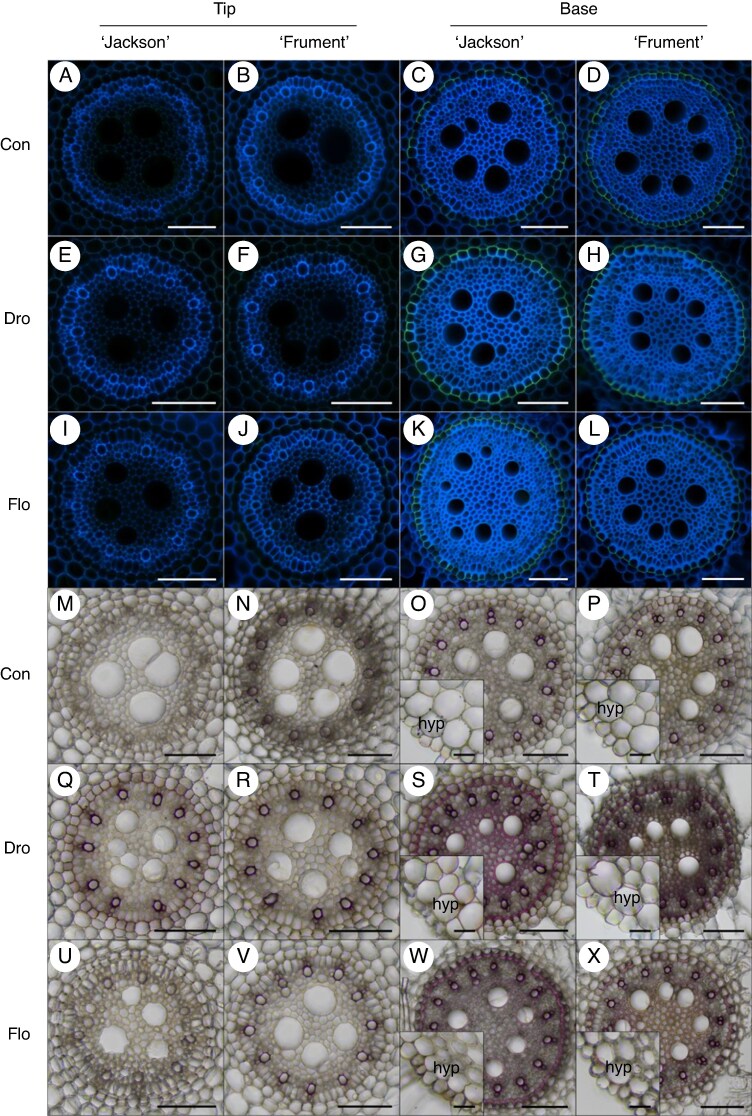
Suberin (A–L) and lignin (M–X) staining of adventitious root cross-sections from the root base and tip of plants of two wheat cultivars (‘Jackson’ and ‘Frument’) grown in control, drought or flooding conditions. (A–L) Epi-fluorescence of stele of cross-sections stained with Fluorol yellow 088, showing green fluorescence from suberin and blue autofluorescence from lignin. (M–X) Whole cross-sections in brightfield, with lignin depositions stained red using Phloroglucinol. Scale bar: 100 µm in overview; 30 µm in detail. Abbreviations: Con, control; Dro, drought; End, endodermis; Flo, flooding; Hyp, hypodermis; Mxy, metaxylem; Pxy, protoxylem; Ste, stele.

Suberin and lignin deposition in the endodermal cell walls respond to water stress. We did not observe suberization of the endodermis in younger root tissues, regardless of cultivar or treatment (Fig. 3), but in older tissues the outer endodermal cell wall was suberized. Water stress resulted in strong suberization of older tissue regardless of cultivar, with almost no passage cells (Fig. 3G, H, K, L). In contrast, the endodermis was incompletely suberized in older tissues of controls with more passage cells (Fig. 3C, D). Likewise, water stress resulted in strong lignification of the endodermis, but also of the stelar tissue and the proto– and metaxylem vessels in older tissues (Fig. 3S, T, W, X). In contrast, the endodermis and metaxylem were not lignified in controls. The degree of lignification of protoxylem vessels in older tissues of controls was similar to the degree of lignification in younger tissues under water stress, indicating early lignification under water stress (Fig. 3O–R, U, V), but lignin deposition was scarce in younger tissues of controls (Fig. 3M, N). Under flooding, there was little lignification in younger tissues of ‘Jackson’, and interestingly, we sporadically found weak lignin deposition in the hypodermis of older tissue under water stress but not in controls (Fig. 3). Overall, water stress markedly enhanced suberization and lignification in older root tissues, whereas younger tissues remained largely unaffected, highlighting stress-induced acceleration of barrier formation and structural reinforcement in wheat roots.

### Altered radial water loss through drought and flooding

**Fig. 4.**
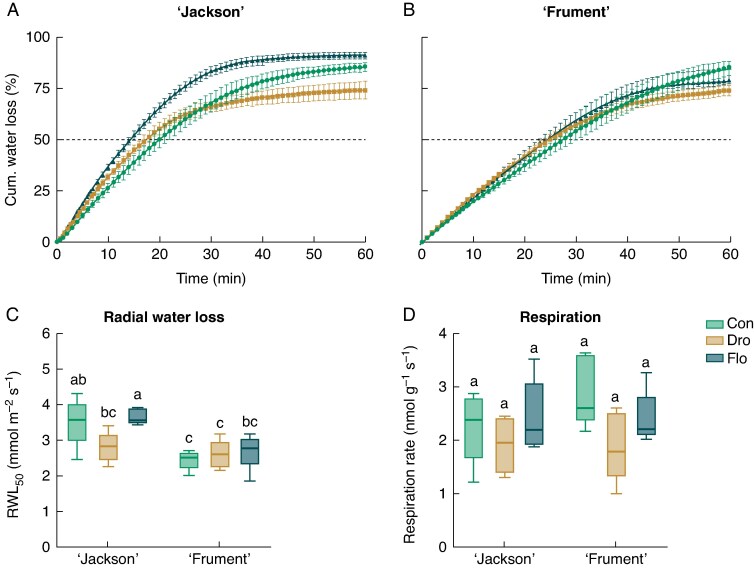
Cumulative water loss from roots of two wheat cultivars [‘Jackson’ (A) and ‘Frument’ (B)], radial water loss at the 50% cumulative water loss threshold (C) and respiration of root segments (D) in control, drought or flooding conditions. (A, B) Values are the mean and SE. (*n* = 5). Dashed line is at 50% cumulative water loss. We compared the radial water loss (Supplementary Data Fig. S5) at the time when 50% of the total tissue water content had evaporated (RWL_50_). (C, D) Tukey’s *post hoc* tests were performed, with different letters representing significant differences. See Table 1 for details on two-way ANOVA results. The box-and-whisker plots display the median (horizontal line), the first and third quartiles (box), and the minimum and maximum values (whiskers). Abbreviations: Con, control; Dro, drought; Flo, flooding.

The RWL has recently been identified as a key indicator of internal tissue barriers to radial water movement (Song *et al.*, 2023). Cumulative water loss in ‘Frument’ was consistently lower in comparison to ‘Jackson’ during the first 40 min of measurement, indicating a higher internal resistance to radial water movement (Fig. 4A, B). Nevertheless, cumulative water loss reached a plateau after 60 min that was similar between cultivars but lower in drought compared with controls. In contrast, cumulative water loss was higher under flooding compared with drought and control conditions in ‘Jackson’; in ‘Frument’, flooding was intermediate between the other two treatments. The RWL declined more rapidly in roots grown in drought and control conditions in ‘Jackson’ than in ‘Frument’ (Supplementary Data Fig. S5). To allow statistical analysis, we compared the RWL at the time when 50% of the total tissue water content had evaporated (RWL_50_), following determination of the Michaelis–Menten constant. Using this approach, ‘Jackson’ had a significantly higher RWL_50_ in control and flooding conditions than ‘Frument’ (Fig. 4C), but RWL_50_ was significantly lower in ‘Jackson’ under drought compared with flooding, a pattern not seen in ‘Frument’. We also measured respiration of root segments 0–25 mm behind the tip to obtain information on the viability of these tissues. In both cultivars, respiration did not change significantly during drought and flooding conditions compared with controls in both cultivars (Fig. 4D).

### Photosynthetic capacity declined under water stress

We measured net photosynthesis (*A*_net_) during the treatments to gain information on the status of the photosynthetic apparatus and carbon fixation, which are influenced by stress conditions. There was a significant effect of time on *A*_net_ in ‘Jackson’ but not in ‘Frument’ (Supplementary Data Fig. S6A, B). Drought caused a stronger negative response in *A*_net_ in both cultivars throughout the experiment compared with flooding. The *A*_net_ declined significantly under drought compared with controls after 12 DOT in ‘Jackson’. At 18 DOT, there was a further decrease in *A*_net_ under drought (total ‘Jackson’, 61% and ‘Frument’, 43%) but also under flooding (total ‘Jackson’, 31% and ‘Frument’, 25 %) in both cultivars compared with controls. The *A*_net_ of controls was stable at 25–30 µmol CO_2_ m^−2^ s^−1^ throughout the measurements in both cultivars.

### Drought and flooding resulted in similar responses of key root traits

**Fig. 5.**
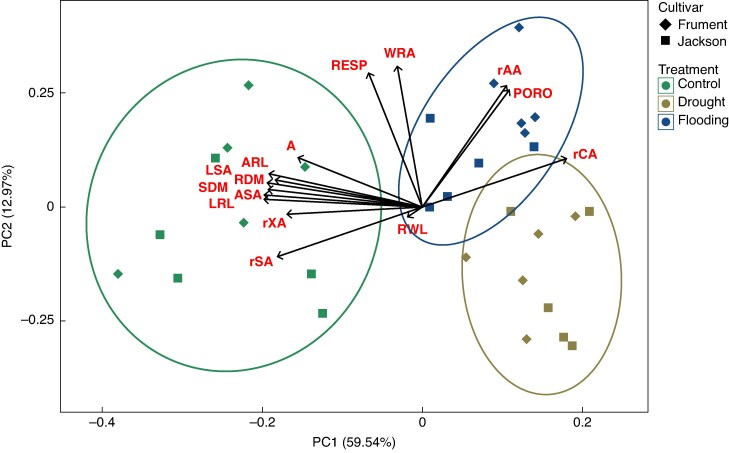
Principal component analysis with selected root traits of two wheat cultivars (‘Jackson’ and ‘Frument’) grown in control (green circles), drought (brown squares) or flooding (blue triangles) conditions. Only normalized data of whole root area and relative cortex, stele, xylem and aerenchyma area from sections 30–40 mm behind the tip were included. In total, PC1 and PC2 explained 73.3% of variance. Abbreviations: A, net photosynthesis (after 18 days of treatment); ARL, adventitious root length; ASA, adventitious root surface area; LRL, lateral root length; LSA, lateral root surface area; PC, principal component; PORO, tissue porosity; rAA, relative aerenchyma area; rCA, relative cortex area; RDM, root dry mass; RESP, respiration; rSA, relative stele area; RWL, radial water loss (50% cumulative water loss threshold); rXA, relative xylem area; SDM, shoot dry mass; WRA, whole root area.

We performed a principal component analysis (PCA), providing an integrated overview of treatment effects that univariate statistical tests failed to detect. Traits of root morphology and anatomy, in addition to *A*_net_ after 18 DOT, were included in the analysis. Principal component 1 (PC1) and principal component 2 (PC2) explained 59.5 and 13.0% of the total variance, respectively (Fig. 5). Traits clearly separated the samples between control and water stress along PC1, which explained the most variance, whereas drought and flooding samples clustered almost completely along PC2, but with much less explanatory power than PC1. Critical parameters causing separation of samples of water stress from controls along PC1 were reduced root length (lateral root length and adventitious root length), surface area (lateral root surface area and adventitious root surface area), DM (root dry mass and shoot dry mass), relative stele and xylem areas, and increased relative cortex area. Tissue porosity and relative aerenchyma area contributed less to PC1. Information from whole root area and respiration was retained in PC2. The RWL did not explain the variance within samples, and PC1 and PC2 did not separate the two cultivars clearly, showing that the two cultivars responded in a similar fashion to contrasting water regimes.

## DISCUSSION

Roots are the first plant organs to be affected by extreme soil water contents, but most studies have focused on drought and flooding in individual studies rather than examining responses to contrasting water regimes in the same experimental set-up (Trnka *et al.*, 2014). In this study, we took a novel approach by simultaneously analysing root responses to both drought and flooding of two wheat cultivars. A hydroponic set-up was used to increase reproducibility and to minimize variability inherent in field studies, such as the unquantifiable adverse effects of soil phytotoxins on plants (Setter *et al.*, 2009). This approach was particularly beneficial for drought treatments, because it allowed for precise maintenance of water potential across the entire root system. We conclude that different underlying physical processes during contrasting water regimes, e.g., water limitation during drought and oxygen deficiency during flooding, result in similar root responses, e.g., increased relative aerenchyma area, lignin and suberin deposition in the endodermis and decreased lateral-to-adventitious root length.

### Drought and flooding impose similar acclimations of root morphology and anatomy

We found that the apical meristems of adventitious roots ceased growth early during both drought and flooding (Fig. 1B). Similar respiration rates, however, indicate that root extension did not cease owing to the death of meristematic and elongating tissues but rather as a consequence of stress perception and signalling (Fig. 4D). The average respiration rate of ‘Frument’ decreased in drought conditions, although not significantly although this trend might have reached statistical significance if the study had included more replicates. Indeed, previous studies have shown early root apical meristem differentiation under drought, and accumulating ethylene negatively affecting root apical meristem proliferation under flooding (Ji *et al.*, 2014; Street *et al.*, 2015). Interestingly, the two cultivars exhibited similar physiological and anatomical acclimations to both drought and flooding.

Both drought and flooding reduced lateral root length and surface area relative to adventitious root length and surface area of both cultivars (Fig. 1C; Supplementary Data Fig. S2B). In control conditions, lateral root proliferation increased the plant–soil interaction surface to optimize nutrient and water uptake. However, under water stress, an extensive root system can have adverse effects, such as increased water requirements, higher desiccation rates under drought (Lynch, 2013 ) or increased radial oxygen loss and phytotoxin uptake under flooding (Colmer *et al.*, 2019). Thus, wheat appears to mitigate these negative consequences by limiting lateral root growth in both stress conditions, highlighting a trade–off between root expansion and stress tolerance.

Our data suggest that the similar early arrest of xylem development is an adaptive trait in wheat in response to drought and flooding, leading to a reduction in XSR and relative xylem area (Fig. 2C; Supplementary Data Fig. S3D). There is an ongoing debate about whether reduced xylem area is associated with a reduced risk of xylem vessel embolism under drought (Lens *et al.*, 2022). In contrast, increased xylem area has been reported in wetland Poaceae species adapted to high soil water content (Yamauchi *et al.*, 2021 ). However, because wheat is a dryland crop that is not acclimatized to flooding, its response to flooding might differ from that of wetland-adapted species, highlighting the inherent difference between adaptation (Yamauchi *et al.*, 2021) and acclimation (present study). The CSR declined to control levels in older tissue under both drought and flooding in comparison to younger tissue (Fig. 2B). This finding was unexpected and remains unexplained owing to limited anatomical data available on the most basal root regions.

Enhancement of physiological barriers through early lignification and suberization of the endodermis is a common response to both drought and flooding, and such response was also confirmed in the present study (Fig. 3). Suberin serves as an apoplastic barrier that limits water and gas diffusion (Franke and Schreiber, 2007). Hence, under drought, suberin deposition can reduce water loss from the stele to the cortex and subsequently into the soil (Zimmermann *et al.*, 2000). Conversely, under flooding, suberization might restrict the influx of toxic dissolved ions from anoxic soils (Franke and Schreiber, 2007). In contrast, the lignification of the endodermis, stelar tissue and metaxylem is considered primarily of structural importance (Soukup *et al.*, 2007; Franke and Schreiber, 2007).

Our PCA revealed distinct clustering among the three treatments (Fig. 5). PC1 separated control samples from water-stressed samples, consistent with data trends indicating a strongly negative response of wheat to water stress. This included root length, surface area, root and shoot DM and relative xylem area, but also *A*_net_. Relative aerenchyma area, in contrast, was scattered along both PC1 and PC2 (i.e. diagonally), suggesting that aerenchyma formation in wheat is not only induced by soil flooding but also by low water potential. Drought and flooding samples separated along PC2; however, this component explained much less variance than PC1 and was therefore of less importance. The PCA identified underlying patterns in the data by integrating multiple variables, which was consistent with univariate statistical analyses; both drought and flooding impaired wheat root growth similarly.

Root and shoot DM decreased significantly under both drought and flooding. However, contrary to our expectations, this reduction did not affect the root–to–shoot ratio, suggesting that neither drought nor flooding resulted in directed allocation of resources to the shoot or root (Fig. 1E; Supplementary Data Fig. S2D, E). Interestingly, these results partly contradict those of a previous study that reported an increase in the root–to–shoot ratio in soil–grown spring wheat under drought (Liu *et al.*, 2004). In fact, enhanced root growth in response to low soil water content is a common strategy for plants to access deeper soil layers with sufficient moisture (Anjum *et al.*, 2017). However, we propose that the lack of a vertical soil moisture gradient in the hydroponic set-up could counteract this typical root growth response.

### Aerenchyma formation and root growth respond similarly to soil flooding in the two cultivars

‘Frument’ was previously found to be more susceptible to prolonged submergence than ‘Jackson’, which was attributed to a more rapidly changing metabolome, as indicated by PCA, and a decline in the integrity of the photosynthetic apparatus (Herzog *et al.*, 2018). Our PCA, which explained 73% of the total variance and included key traits of root morphology, anatomy and plant growth, did not reveal clear differences between the two cultivars in terms of waterlogging (Fig. 5). It is plausible that ‘Frument’ is more susceptible to flooding (either waterlogging or submergence) at different phenological stages, such as anthesis or grain–filling, because the severity of flooding on plant health and growth depends on the timing and duration of the stress within the developmental context (Ploschuk *et al.*, 2018, 2020). However, the physiological and anatomical responses of ‘Frument’ under flooding do not suggest a greater susceptibility to water stress compared with ‘Jackson’ in this study.

Roots of ‘Jackson’ desiccated faster than those of ‘Frument’ under flooding, suggesting cultivar-specific variations in desiccation dynamics (Fig. 4A, B). According to modelling, RWL decreases with increasing root diameter, decreasing tissue porosity and the presence of a suberized and lignified exodermis (Song *et al.*, 2023 ). Thicker roots have a lower surface area-to-volume ratio, which reduces oxygen loss to anoxic soils and limits RWL (Pedersen *et al.*, 2021). Consequently, thicker roots might counteract the increased RWL associated with aerenchyma formation, which is necessary to enhance longitudinal oxygen movement during soil flooding (Song *et al.*, 2023). However, in our study, root diameter and porosity do not explain the observed differences in RWL among cultivars. According to our PCA, RWL contributed insignificantly to the variance between treatments and cultivars, indicating that the observed differences in RWL might not be closely linked to drought or flooding tolerance in wheat with the present experimental approach.

### Conclusions and outlook

Although anatomical responses, such as aerenchyma formation, offer adaptive advantages to both water stresses, the underlying physical processes, e.g., water limitation during drought and oxygen deficiency during flooding, driving these responses differ between drought and flooding conditions. Drought restricted both the maximum length and the growth rate of individual adventitious roots, in addition to the total length and surface area of the entire root system, closely mirroring flooding conditions. Anatomical features, i.e. CSR, XSR, ACR, and lignin and suberin deposition, also reacted in a similar manner to drought and flooding. We conclude that various root morphological and anatomical features of the two wheat cultivars evaluated acclimate similarly to both drought and flooding. Unlike Herzog *et al.* (2018) in his submergence study, we do not report differences between ‘Jackson’ and ‘Frument’ under water stress. Future research should therefore extend this comparison to other wheat organs to provide a more comprehensive understanding of cross-stress effects on root morphology, anatomy and physiology. Additionally, an important avenue for exploration is whether pre-exposure to one form of water stress can prime wheat for enhanced resilience to subsequent, contrasting stress conditions, because this could inform breeding programmes aimed at developing wheat cultivars with outstanding performance in both drought and flooding scenarios.

## Supplementary Material

mcaf152_Supplementary_Data

## SUPPLEMENTARY DATA

Supplementary data are available at *Annals of Botany* online and consist of the following. Table S1: key root traits versus source of variation, percentage of total variation explained by each variable and the *P*-value for two-way ANOVA. Figure S1: linear regression of root length of wheat grown grown in control, drought and flooding conditions. Figure S2: ratio of lateral and adventitious root surface area, lateral root length, total surface area, shoot dry mass and root-to-shoot ratio. Figure S3: whole root area and relative cortex, stele, xylem and aerenchyma area of adventitious root cross-sections. Figure S4: tissue porosity. Figure S5: radial water loss. Figure S6: net photosynthesis.

## AI ASSISTANCE

AI was used for minor text improvements and proof-reading. AI was used to facilitate coding in R.
