# Supplementary material for: Root morphology and anatomy respond similarly to drought and flooding in two wheat cultivars
Source: Ann Bot. 2025 Jul 22;136(5-6):1239–50. doi: 10.1093/aob/mcaf152 (PMC12682829; doi:10.1093/aob/mcaf152)
Supplement: mcaf152_Supplementary_Data [file mcaf152_supplementary_data.docx]

# Supplementary Information for:

## Root morphology and anatomy respond similarly to drought and flooding in two wheat cultivars

Tobias **Guhr^1,2^***, Zhiwei **Song^1^**, Albert **G. Andersen^1^**, Juan de la Cruz **Jiménez^1^** and Ole **Pedersen^1,3^***

1) Department of Biology, University of Copenhagen, Universitetsparken 4, 3rd floor, 2100 Copenhagen, Denmark 2) University of Bonn, Regina-Pacis-Weg 3, 53113 Bonn, Germany 3) School of Biological Sciences, The University of Western Australia, 35 Stirling Highway, Crawley, WA 6009, Australia

*Ole Pedersen: [opedersen@bio.ku.dk](mailto:opedersen@bio.ku.dk); Tobias Guhr: [s6toguhr@uni-bonn.de](mailto:s6toguhr@uni-bonn.de)

## List of supplementary tables and figure

**Table S1** Key root trait *versus* source of variation, percentage of total variation explained by each variable, and the p value for two-way ANOVA.

**Fig. S1:** Linear regression of root length of wheat grown grown under control (green), drought (brown) and flooding (blue) conditions.

**Fig. S2:** Ratio of lateral and adventitious root surface area (A), lateral root length (B), total root surface area (C), shoot dry mass (D) and root-to-shoot ratio (E) of two wheat cultivars (‘Jackson’ and ‘Frument’) grown under control (green), drought (brown) or flooding (blue) conditions.

**Fig. S3:** Whole root area (A) and relative cortex (B), stele (C), xylem (D) and aerenchyma (E) area of adventitious root cross-sections from the base (dark colours) and from the tip (light colours) of two wheat cultivars (‘Jackson’ and ‘Frument’).

**Fig. S4:** Tissue porosity of wheat cultivars (‘Jackson’ and ‘Frument’) under control (green), drought (brown) or flooding (blue) conditions.

**Fig. S5:** Radial water loss of two wheat cultivars ‘Jackson’ (A) and ‘Frument’ (B) under control (green), drought (brown) or flooding (blue) conditions.

**Fig. S6:** Net photosynthesis of two wheat cultivars ‘Jackson’ (right) and ‘Frument’ (left) grown under control (green), drought (brown) or flooding (blue) conditions.

**Table S1** Key root trait *versus* source of variation, percentage of total variation explained by each variable, and the p value for two-way ANOVA. P values > 0.05 are not shown (n.s.).

| **Variable** | **Source of variation** | **% of total variation** | **p value** |
| --- | --- | --- | --- |
| Lateral root length  Fig. S2 | Cultivar | 0 | n.s. |
|  | Treatment | 86.5 | < 0.0001 |
|  | Cultivar × treatment | 2.6 | n.s. |
|  | *Residual* | 10.9 |  |
| Root surface area ratio  Fig. S2 | Cultivar | 2.6 | n.s. |
|  | Treatment | 44.9 | < 0.001 |
|  | Cultivar × treatment | 2.0 | n.s. |
|  | *Residual* | 50.5 |  |
| Total surface area  Fig. S2 | Cultivar | 0.1 | n.s. |
|  | Treatment | 74.0 | < 0.0001 |
|  | Cultivar × treatment | 2.9 | n.s. |
|  | *Residual* | 23.0 |  |
| Shoot dry mass  Fig. S2 | Cultivar | 0.1 | n.s. |
|  | Treatment | 78.6 | < 0.0001 |
|  | Cultivar × treatment | 5.1 | 0.03850 |
|  | *Residual* | 16.3 |  |
| Root-to-shoot ratio  Fig. S2 | Cultivar | 1.3 | n.s. |
|  | Treatment | 43.0 | < 0.001 |
|  | Cultivar × treatment | 9.7 | n.s. |
|  | *Residual* | 46.0 |  |
| Root cross-sectional area  cv. ‘Jackson’  Fig. S3 | Position | 25.3 | < 0.001 |
|  | Treatment | 27.4 | < 0.01 |
|  | Position × treatment | 5.8 | n.s. |
|  | *Residual* | 41.6 |  |
| Root cross-sectional area  cv. ‘Frument’  Fig. S3 | Position | 23.2 | < 0.001 |
|  | Treatment | 30.5 | < 0.001 |
|  | Position × treatment | 12.9 | 0.01963 |
|  | *Residual* | 33.4 |  |
| Relative cortex area  cv. ‘Jackson’  Fig. S3 | Position | 36.0 | < 0.001 |
|  | Treatment | 9.4 | n.s. |
|  | Position × treatment | 8.3 | n.s. |
|  | *Residual* | 46.3 |  |
| Relative cortex area  cv. ‘Frument’  Fig. S3 | Position | 57.8 | < 0.0001 |
|  | Treatment | 16.2 | < 0.001 |
|  | Position × treatment | 6.5 | 0.0311 |
|  | *Residual* | 19.5 |  |
| Relative stele area  cv. ‘Jackson’  Fig. S3 | Position | 36.0 | < 0.001 |
|  | Treatment | 9.4 | n.s. |
|  | Position × treatment | 8.3 | n.s. |
|  | *Residual* | 46.3 |  |
| Relative stele area  cv. ‘Frument’  Fig. S3 | Position | 57.8 | < 0.0001 |
|  | Treatment | 16.2 | < 0.001 |
|  | Position × treatment | 6.5 | 0.0311 |
|  | *Residual* | 19.5 |  |
| Relative xylem area  cv. ‘Jackson’  Fig. S3 | Position | 3.8 | 0.0270 |
|  | Treatment | 78.7 | < 0.0001 |
|  | Position × treatment | 1.3 | n.s. |
|  | *Residual* | 16.2 |  |
| Relative xylem area  cv. ‘Frument’  Fig. S3 | Position | 1.4 | n.s. |
|  | Treatment | 74.2 | < 0.0001 |
|  | Position × treatment | 3.6 | n.s. |
|  | *Residual* | 20.8 |  |
| Relative aerenchyma area  cv. ‘Jackson’  Fig. S3 | Position | 10.7 | 0.0124 |
|  | Treatment | 50.7 | < 0.0001 |
|  | Position × treatment | 3.6 | n.s. |
|  | *Residual* | 35.1 |  |
| Relative aerenchyma area  cv. ‘Frument’  Fig. S3 | Position | n.a. | n.s. |
|  | Treatment | n.a. | < 0.01 |
|  | Position × treatment | n.a. | < 0.001 |
| Tissue porosity  Fig. S4 | Cultivar | 6.9 | 0.0319 |
|  | Treatment | 60.3 | < 0.0001 |
|  | Cultivar × treatment | 1.0 | n.s. |
|  | *Residual* | 31.8 |  |
| Net photosynthesis  12 DOT  Fig. | Cultivar | 0.7 | n.s. |
|  | Treatment | 29.4 | < 0.01 |
|  | Cultivar × treatment | 11.9 | n.s. |
|  | *Residual* | 58.1 |  |
| Net photosynthesis  18 DOT  Fig. | Cultivar | 1.4 | n.s. |
|  | Treatment | 68.6 | < 0.0001 |
|  | Cultivar × treatment | 2.0 | n.s. |
|  | *Residual* | 28.0 |  |

**
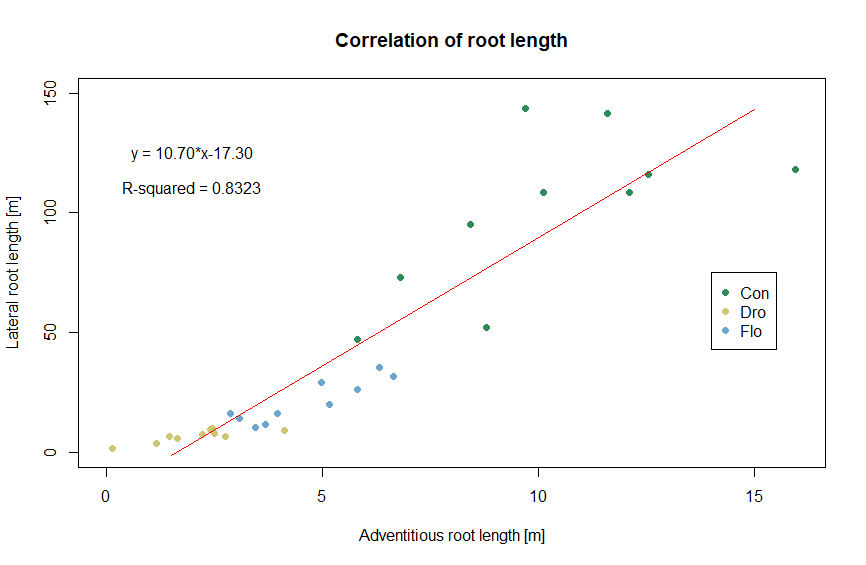
Fig. S1: Linear regression of root length of wheat grown grown under control (green), drought (brown) and flooding (blue) conditions.** Equation and r-value of linear regression are shown.


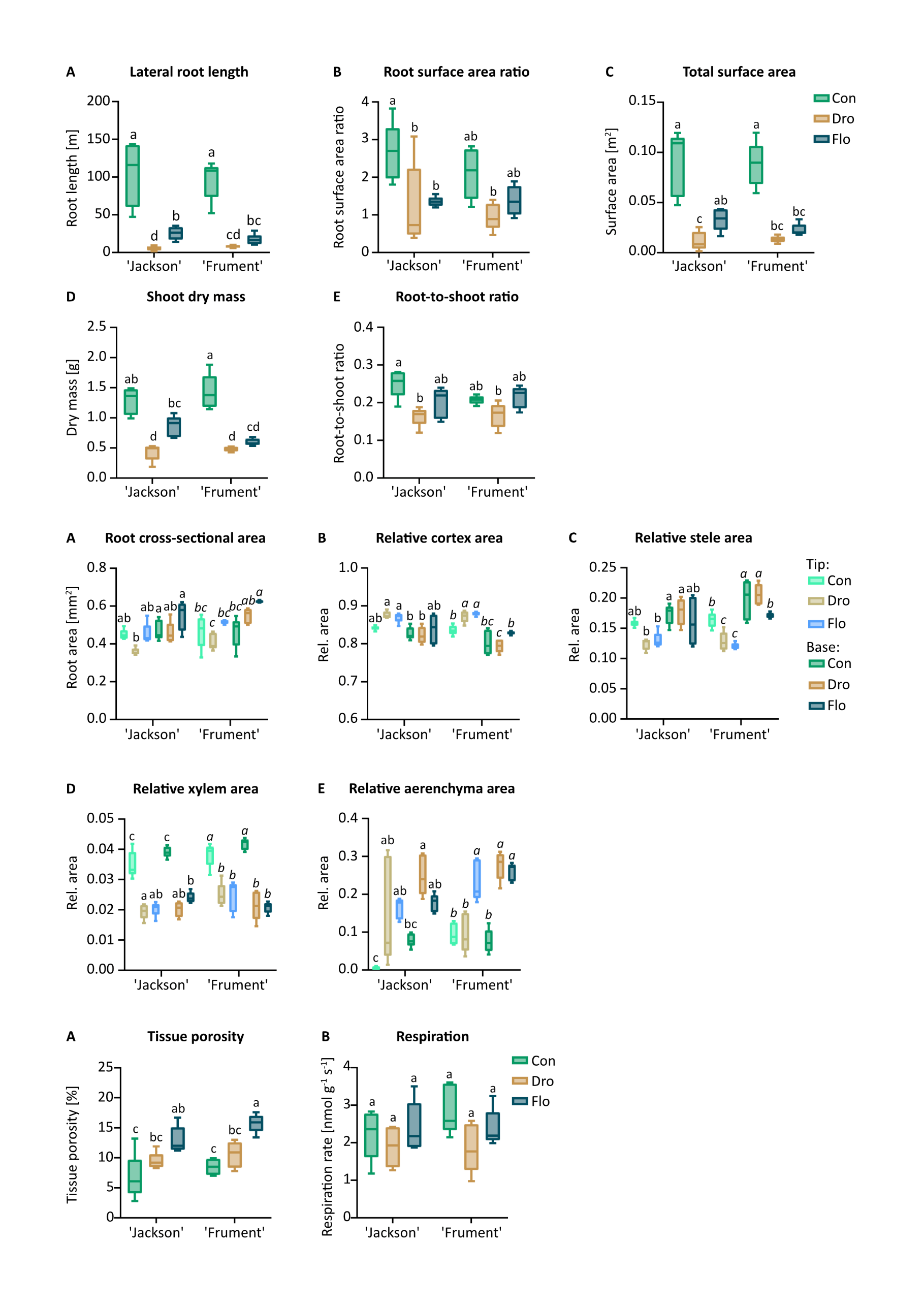


**Fig. S2: Ratio of lateral and adventitious root surface area (A), lateral root length (B), total root surface area (C), shoot dry mass (D) and root-to-shoot ratio (E) of two wheat cultivars (‘Jackson’ and ‘Frument’) grown under control (green), drought (brown) or flooding (blue) conditions.** Growth before stress exposure was excluded by subtracting values obtained from plants harvested prior start of the treatments. **A‑C** Roots with a root diameter below 0.5 mm were classified as laterals and above 0.5 mm as adventitious roots (n=5). In A, lateral root surface area was divided by adventitious root surface area. **E** Root dry mass (main Fig. 1E) was divided by shoot dry mass (Supp Fig. S2D). **A-E** Tukey post-hoc tests were performed with different letters representing significant differences. See Table S1 for details on 2-way ANOVA results. Con = Control; Dro = Drought; Flo = Flooding.


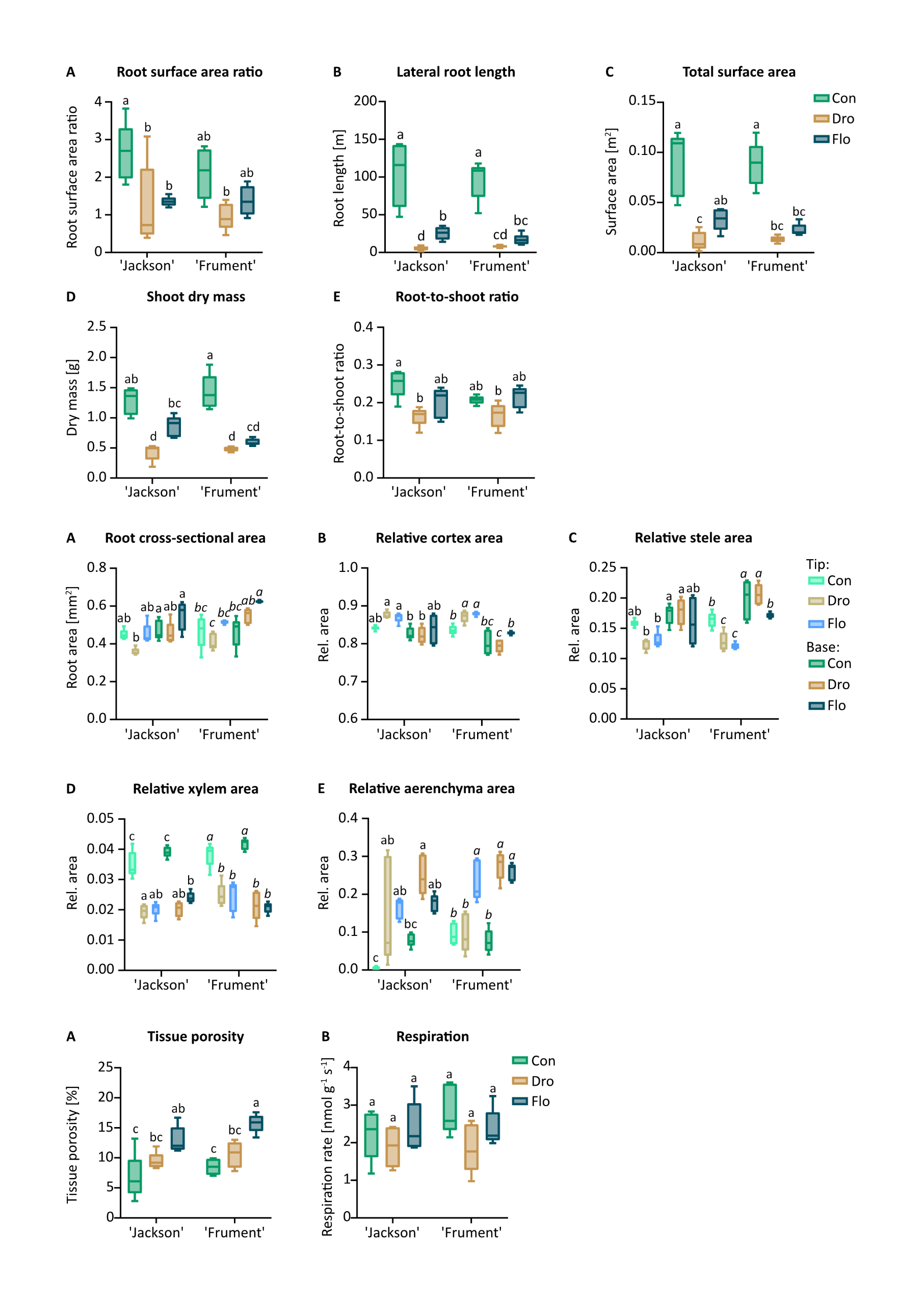
**Fig. S3: Whole root area (A) and relative cortex (B), stele (C), xylem (D) and aerenchyma (E) area of adventitious root cross-sections from the base (dark colours) and from the tip (light colours) of two wheat cultivars (‘Jackson’ and ‘Frument’).** Two wheat cultivars were grown under control (green), drought (brown) or flooding (blue) conditions. The two positions represent younger (30-40 mm the root tip) and older (base region, 0-10 mm behind the root-shoot junction) tissue. Adventitious roots were sampled and sectioned after 20 days of treatment. **B-E** Tissue areas relative to whole root cross-sectional area (A). Tukey post-hoc tests were performed for each cultivar separately with different letters representing significant differences. See Table S1 for details on 2-way ANOVA results. Con = Control; Dro = Drought; Flo = Flooding.

**
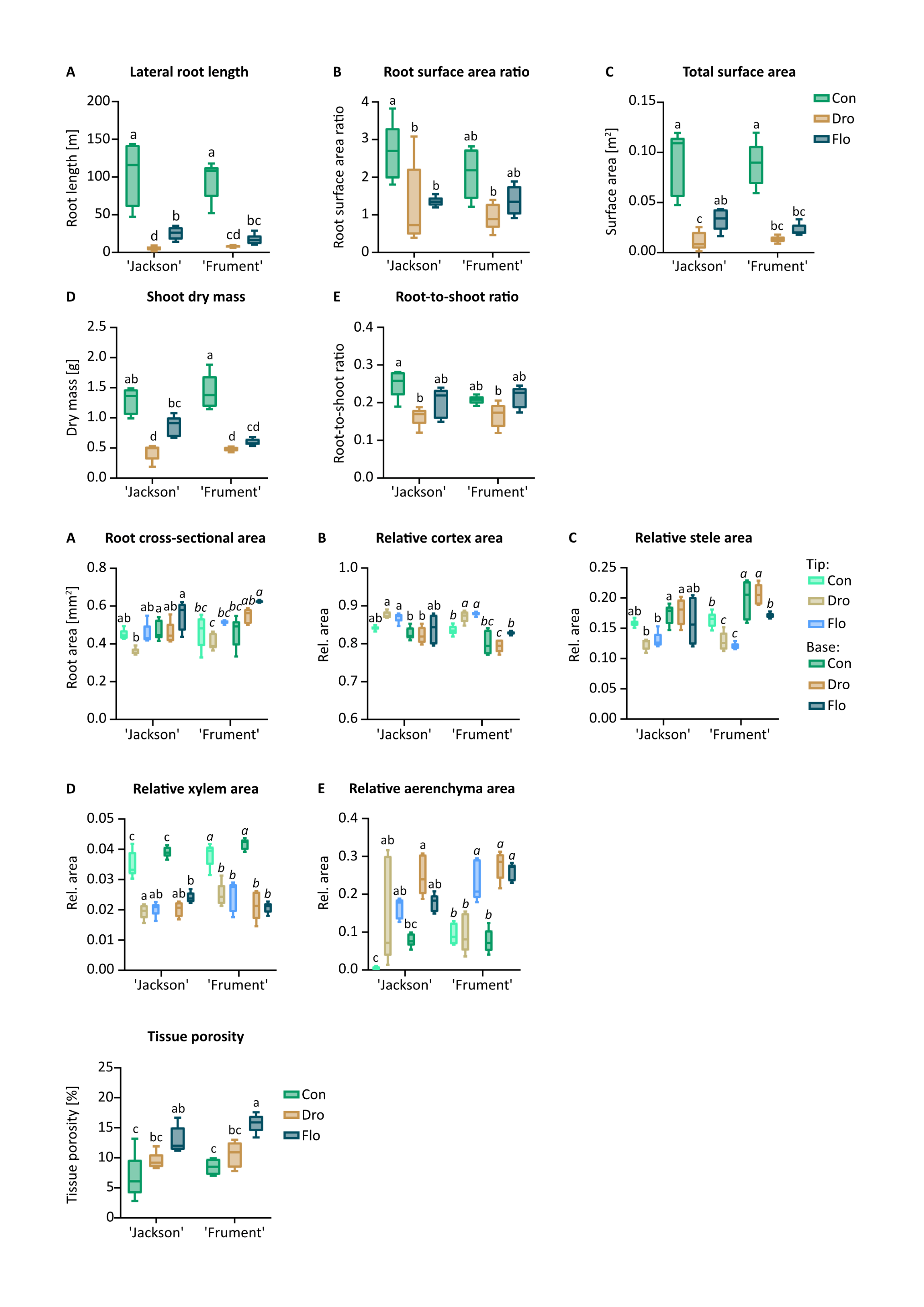
Fig. S4: Tissue porosity of wheat cultivars (‘Jackson’ and ‘Frument’) under control (green), drought (brown) or flooding (blue) conditions.** Tissue porosity comprises both aerenchyma and intercellular air spaces. Tukey post-hoc tests were performed with different letters representing significant differences. See Table S1 for details on 2-way ANOVA results. Con = Control; Dro = Drought; Flo = Flooding.


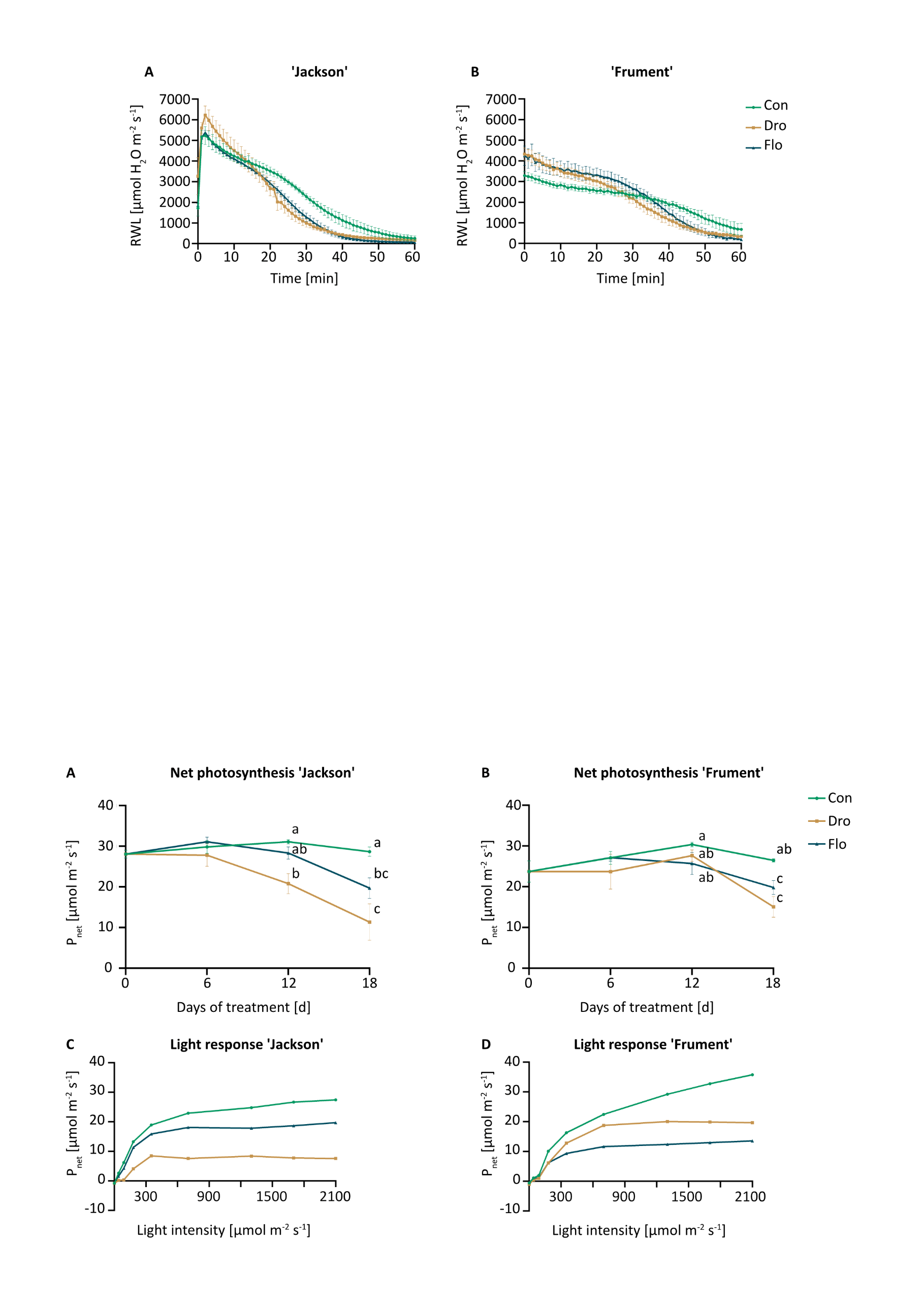
**Fig. S5: Radial water loss of two wheat cultivars ‘Jackson’ (A) and ‘Frument’ (B) under control (green), drought (brown) or flooding (blue) conditions.** Values are mean and standard error (n=5). Con = Control; Dro = Drought; Flo = Flooding.

**
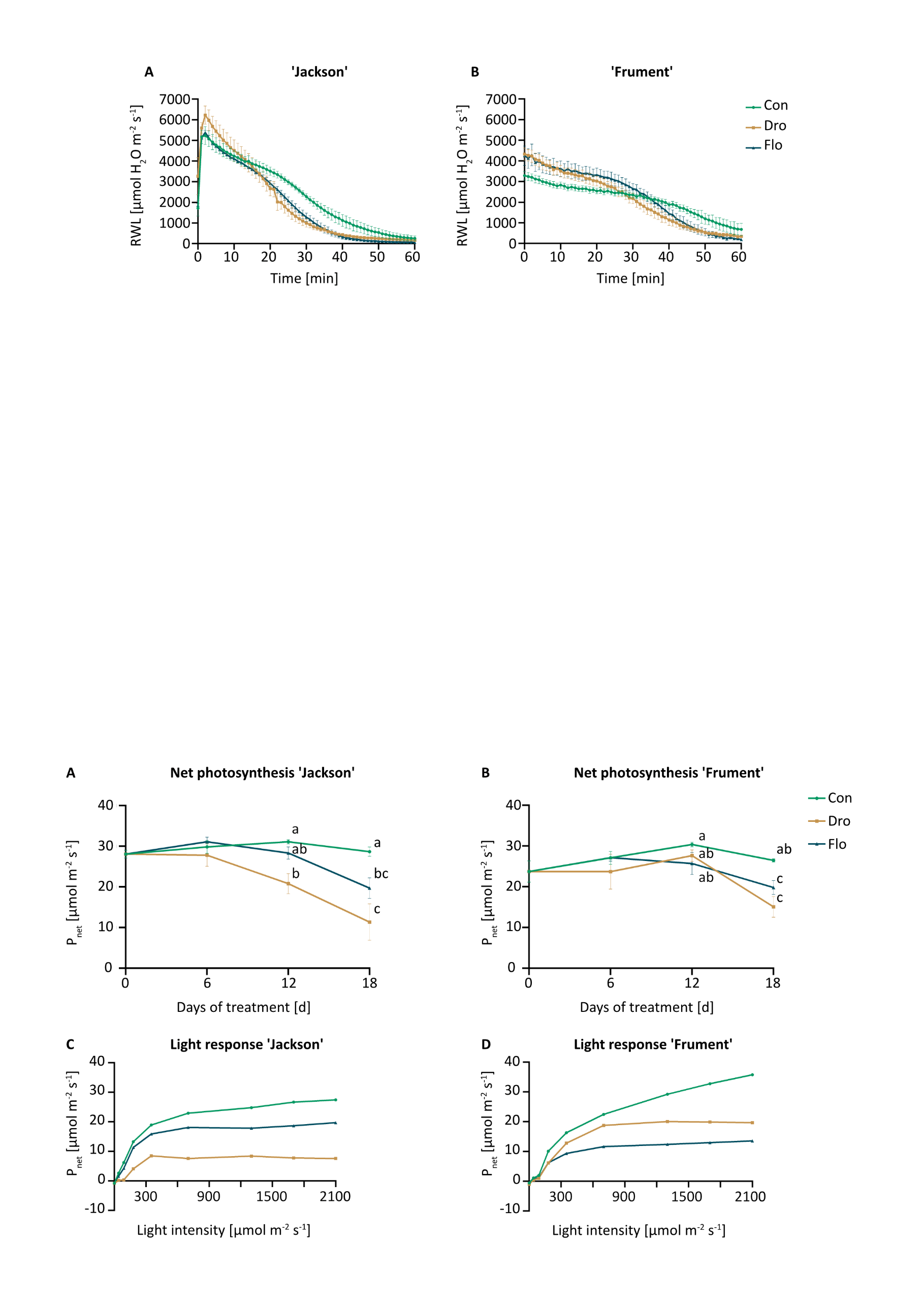
Fig. S6: Net photosynthesis of two wheat cultivars ‘Jackson’ (right) and ‘Frument’ (left) grown under control (green), drought (brown) or flooding (blue) conditions. A-B** Values are mean and standard error (n=5). The second youngest fully expanded leaf was measured. Significant differences between cultivars and treatments on each time point were tested using Tukey post-hoc test. Different letters represent significant differences. Letters for days 0 and 6 of treatment are not shown since there are no significant differences. See Table S1 for details on 2-way ANOVA results. Con = Control; Dro = Drought; Flo = Flooding.
